# Supplementary material for: Childhood brain tumors instruct cranial hematopoiesis and immunotolerance
Source: Nat Genet. 2026 Feb 3;58(2):317–28. doi: 10.1038/s41588-025-02499-2 (PMC12900635; doi:10.1038/s41588-025-02499-2)
Supplement: Supplementary file 1 — Supplementary Figs. 1–10. [file 41588_2025_2499_MOESM1_ESM.pdf]

nature genetics

Supplementary information https://doi.org/10.1038/s41588-025-02499-2

Childhood brain tumors instruct cranial hematopoiesis and immunotolerance

In the format provided by the authors and unedited

Supplementary Figures:

Supplementary Fig. 1 Autochthonous Nestin^{CreERT2};Nestin$^{Flx\text{-}STOP\text{-}FlxZFTA\text{-}RELA}$ mouse model of ZFTA-RELA fusion-driven ependymoma

Supplementary Fig. 2 Representative flow cytometry gating strategies.

Supplementary Fig. 3 Integration of PTPRC^{+} cells from human foetal brain and childhood brain tumour single-cell RNA sequencing datasets.

Supplementary Fig. 4 Human choroid plexus carcinoma neurosurgical CNS immune tissue reveals HLA-DR-expressing haematopoietic stem progenitor cells (HSPCs) in skull, dura and tumour parenchyma.

Supplementary Fig. 5 Local increased proliferation of skull bone marrow cells in EP$^{ZFTA\text{-}RELA}$**-bearing mice, which is absent in peripheral bone marrow niches.**

Supplementary Fig. 6 Experimental pipeline and cell type annotation of single-cell RNA-sequencing of mouse neuroimmune tissues in EP$^{ZFTA\text{-}RELA}$**-bearing and control bearing mice.**

Supplementary Fig. 7 CSF access and modulate skull bone marrow niche in EP$^{ZFTA\text{-}RELA}$**-bearing mice.**

Supplementary Fig. 8 Combined analysis of chromatin accessibility and gene expression in HSCs from skull and tibia of EP$^{ZFTA\text{-}RELA}$**-bearing and control-bearing mice.**

Supplementary Fig. 9 Single-cell RNA-sequencing highlights CSFR2A expression on skull and dura in EP$^{ZFTA\text{-}RELA}$-bearing mice

Supplementary Fig. 10. Increased T cell infiltrate in single-cell RNAseq of intratumoural immune compartment following treatment with mavrilimumab in EP$^{ZFTA\text{-}RELA}$ mice.

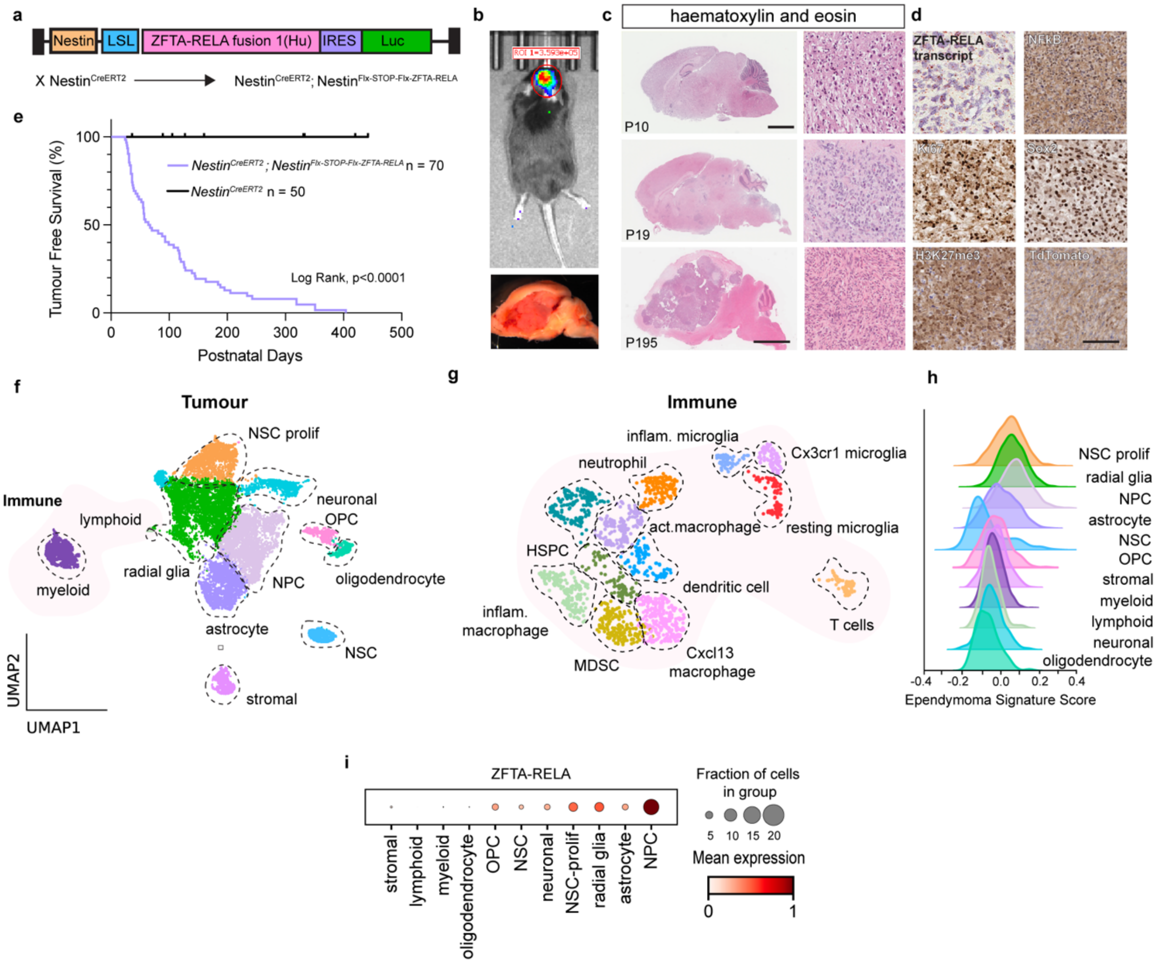
a
Nestin
LSL
ZFTA-RELA fusion 1(Hu)
IRES
Luc
X NestinCreERT2
NestinCreERT2; NestinFlx-STOP-Flx-ZFTA-RELA
b
c
haematoxylin and eosin
P10
P19
P195
d
ZFTA-RELA transcript
NFkB
Ki67
Sox2
H3K27me3
TdTomato
e
Tumour Free Survival (%)
NestinCreERT2; NestinFlx-STOP-Flx-ZFTA-RELA n = 70
NestinCreERT2 n = 50
Log Rank, p<0.0001
Postnatal Days
f
Tumour
Immune
NSC prolif
neuronal
OPC
oligodendrocyte
lymphoid
myeloid
radial glia
NPC
astrocyte
NSC
stromal
UMAP2
UMAP1
g
Immune
inflam. microglia
Cx3cr1 microglia
neutrophil
act.macrophage
resting microglia
HSPC
dendritic cell
T cells
inflam. macrophage
MDSC
Cxcl13 macrophage
h
NSC prolif
radial glia
NPC
astrocyte
NSC
OPC
stromal
myeloid
lymphoid
neuronal
oligodendrocyte
Ependymoma Signature Score
i
ZFTA-RELA
Fraction of cells in group
Mean expression
stromal
lymphoid
myeloid
oligodendrocyte
OPC
NSC
neuronal
NSC-prolif
radial glia
astrocyte
NPC

Supplementary Fig. 1 Autochthonous Nestin^{CreERT2};Nestin$^{Flx\text{-}STOP\text{-}FlxZFTA\text{-}RELA}$ mouse model of ZFTA-RELA fusion-driven ependymoma. a, Schematic of lox-stop-lox cassette and breeding strategy for the generation of the Nestin^{CreERT2};Nestin$^{Flx\text{-}STOP\text{-}FlxZFTA\text{-}RELA}$ mouse model. **B** Representative bioluminecense and TdTomato signal of 28 day old EP$^{ZFTA\text{-}RELA}$ mice. **c,** Representative haematoxylin and Eosin staining of endpoint tumour. **d,** Representative immunohistochemical staining of Nestin^{CreERT2};Nestin$^{Flx\text{-}STOP\text{-}FlxZFTA\text{-}RELA}$ mouse tumours stained for canonical ZFTA-RELA ependymoma markers**. e**, **,** Kaplein Meier survival of Nestin^{CreERT2};Nestin$^{Flx\text{-}STOP\text{-}FlxZFTA\text{-}RELA}$ mouse model (n = 70), Log-rank MantelCox test relative to Nestin^{CreERT2} control mice (n = 50), median survival 92 ± 16 days. **f** uniform manifold approximation and projection (UMAP) visualisation of single-cell RNAseq of tumour parenchyma of EP$^{ZFTA\text{-}RELA}$ mice (n = 4 mice), coloured by cell-type annotations; neural stem cell (NSC) proliferating (prolif.) radlial glia, neural precursor cell (NPC), astrocyte, NSC, oligodendrocyte precursor cell (OPC), stromal, myeloid, lymphoid, neuronal and oligodendrocyte. **g** UMAP visualisation of single-cell RNAseq of tumour tumour parenchyma of EP$^{ZFTA\text{-}RELA}$ mice (n = 4 mice), coloured by secondary cell-type annotations of immune populations; inflammatory (inflam.) miroglia, Cx3cr1 microglia, resting microglia, T cells, Cxcl13 macrophage, dendritic cell, activated (act.) macrophage, neutrophil, haematopoietic stem progenitor cell (HSPC), inflammatory macrophage and myeloid-derived suppressor cell (MDSC) **h**, enrichment plot of ependymoma signature score across cell types. **i**, Dotplot of proportion of and mean expression of ZFTA-RELA gene across cell types.

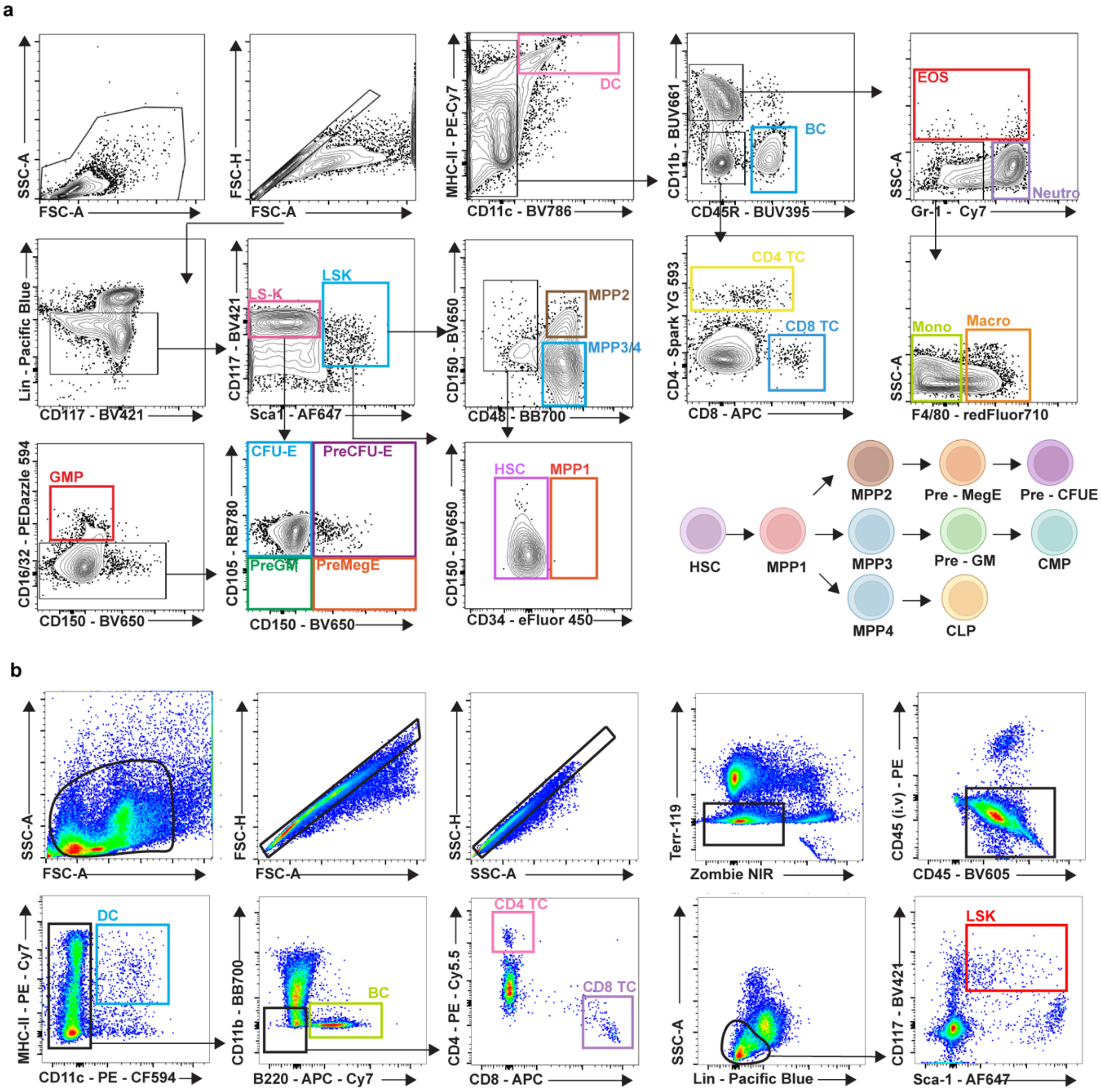
a
SSC-A
FSC-A
FSC-H
FSC-A
MHC-II - PE-Cy7
CD11c - BV786
DC
CD11b - BUV661
CD45R - BUV395
BC
SSC-A
Gr-1 - Cy7
EOS
Neutro
Lin - Pacific Blue
CD117 - BV421
CD117 - BV421
Sca1 - AF647
LS-K
LSK
CD150 - BV650
CD48 - BB700
MPP2
MPP3/4
CD4 - Spark YG 593
CD8 - APC
CD4 TC
CD8 TC
SSC-A
F4/80 - redFluor710
Mono
Macro
CD16/32 - PEDazzle 594
CD150 - BV650
GMP
CD105 - RB780
CD150 - BV650
CFU-E
PreCFU-E
PreGM
PreMegE
CD150 - BV650
CD34 - eFluor 450
HSC
MPP1
HSC
MPP1
MPP2
Pre - MegE
Pre - CFUE
MPP3
Pre - GM
CMP
MPP4
CLP
b
SSC-A
FSC-A
FSC-H
FSC-A
SSC-H
SSC-A
Terr-119
Zombie NIR
CD45 (i.v) - PE
CD45 - BV605
MHC-II - PE - Cy7
CD11c - PE - CF594
DC
CD11b - BB700
B220 - APC - Cy7
BC
CD4 - PE - Cy5.5
CD8 - APC
CD4 TC
CD8 TC
SSC-A
Lin - Pacific Blue
CD117 - BV421
Sca-1 - AF647
LSK

Supplementary Fig. 2 Representative flow cytometry gating strategies. a, Gating strategy for the annotation of haematopoietic stem progenitor cell (HSPC) populations in murine tissue; multipotent progenitor (MPP)3/4, MPP2, MPP1, haematopoietic stem cell (HSC), granulocyte-monocyte precursor (GMP) pre-megakaryocyte/erythrocyte (PreMegE), pre- granulocyte monocyte (preGM), pre-colony-forming unit-erythroid cell (CFU-E), CFU-E, LSK-, CD8 T cell (TC), CD4 TC, neutrophil (Neutro.), monocyte (Mono.), macrophage (Macro.) eosinophil (EOS), B cell (BC) and dendritic cell (DC). **b**, Gating strategy for fluorescence-activated cell sorting (FACS) of immune cells for antigen presentation assays.

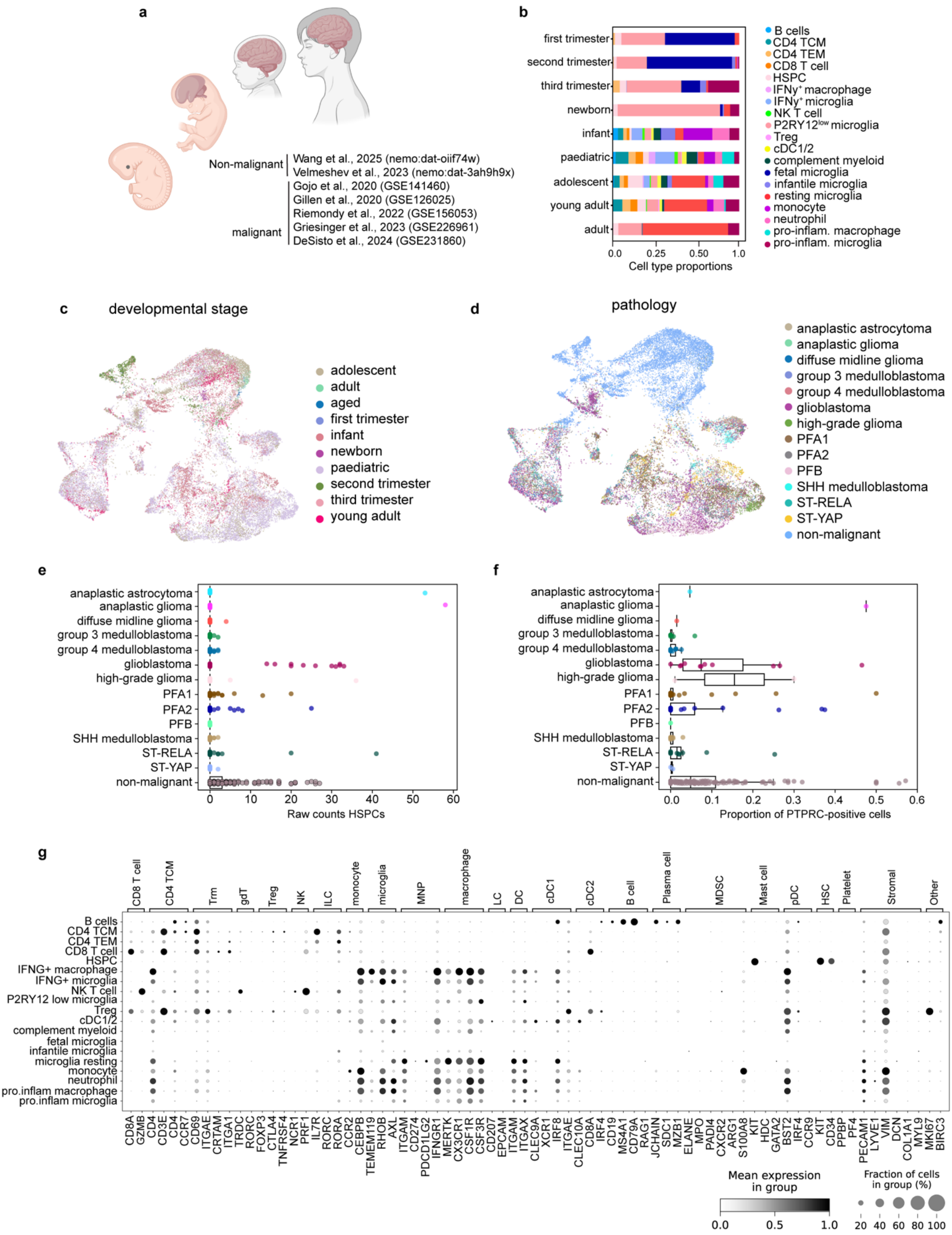

a
Non-malignant
Wang et al., 2025 (nemo:dat-oiif74w)
Velmeshev et al., 2023 (nemo:dat-3ah9h9x)
Gojo et al., 2020 (GSE141460)
Gillen et al., 2020 (GSE126025)
Riemondy et al., 2022 (GSE156053)
Griesinger et al., 2023 (GSE226961)
DeSisto et al., 2024 (GSE231860)
malignant
b
first trimester
second trimester
third trimester
newborn
infant
paediatric
adolescent
young adult
adult
0.0
0.25
0.50
0.75
1.0
Cell type proportions
B cells
CD4 TCM
CD4 TEM
CD8 T cell
HSPC
IFNγ+ macrophage
IFNγ+ microglia
NK T cell
P2RY12low microglia
Treg
cDC1/2
complement myeloid
fetal microglia
infantile microglia
resting microglia
monocyte
neutrophil
pro-inflam. macrophage
pro-inflam. microglia
c
developmental stage
adolescent
adult
aged
first trimester
infant
newborn
paediatric
second trimester
third trimester
young adult
d
pathology
anaplastic astrocytoma
anaplastic glioma
diffuse midline glioma
group 3 medulloblastoma
group 4 medulloblastoma
glioblastoma
high-grade glioma
PFA1
PFA2
PFB
SHH medulloblastoma
ST-RELA
ST-YAP
non-malignant
e
anaplastic astrocytoma
anaplastic glioma
diffuse midline glioma
group 3 medulloblastoma
group 4 medulloblastoma
glioblastoma
high-grade glioma
PFA1
PFA2
PFB
SHH medulloblastoma
ST-RELA
ST-YAP
non-malignant
0
10
20
30
40
50
60
Raw counts HSPCs
f
0.0
0.1
0.2
0.3
0.4
0.5
0.6
Proportion of PTPRC-positive cells
g
CD8 T cell
CD4 TCM
Trm
gdT
Treg
NK
ILC
monocyte
microglia
MNP
macrophage
LC
DC
cDC1
cDC2
B cell
Plasma cell
MDSC
Mast cell
pDC
HSC
Platelet
Stromal
Other
B cells
CD4 TCM
CD4 TEM
CD8 T cell
HSPC
IFNG+ macrophage
IFNG+ microglia
NK T cell
P2RY12 low microglia
Treg
cDC1/2
complement myeloid
fetal microglia
infantile microglia
microglia resting
monocyte
neutrophil
pro.inflam macrophage
pro.inflam microglia
CD8A
GZMB
CD4
CD3E
CD4
CCR7
CD69
ITGAE
CRTAM
ITGA1
TRDC
RORC
FOXP3
CTLA4
TNFRSF4
NCR1
PRF1
IL7R
RORC
RORA
CCR2
CEBPB
TEMEM119
RHOB
AXL
ITGAM
CD274
PDCD1LG2
IFNGR1
MERTK
CX3CR1
CSF1R
CSF3R
CD207
EPCAM
ITGAM
ITGAX
CLEC9A
XCR1
IRF8
ITGAE
CLEC10A
CD8A
IRF4
CD19
MS4A1
CD79A
RAG1
JCHAIN
SDC1
MZB1
ELANE
MPO
PADI4
CXCR2
ARG1
S100A8
KIT
HDC
GATA2
BST2
IRF4
CCR9
KIT
CD34
PPBP
PF4
PECAM1
LYVE1
VIM
DCN
COL1A1
MYL9
MKI67
BIRC3
Mean expression in group
0.0
0.5
1.0
Fraction of cells in group (%)
20
40
60
80
100

Supplementary Fig. 3 Integration of PTPRC$^+$ cells from human foetal brain and childhood brain tumour single-cell RNA sequencing datasets. a, schematic summarising the sourcing of previously published datasets created in biorender.com. **b**, stacked bar plot of the proportion of annotated cell types in single-cell RNAseq of integrated from human fetal brain and childhood brain tumour PTPRC$^+$ cells, across development stages; B cells CD4 tissue central memory (TCM), CD4 Tissue effector memory (TEM). CD8 T cell, HSPCs, interferon-gamma (IFN-y) macrophages, IFN-y microglia, natural killer (NK) T cell, Purinergic Receptor P2Y12 (P2RY12) low microglia, Treg, conventional dendritic cell (cDC) 1/2 complement myeloid, foetal, infantile and resting microglia, monocyte, neutrophil and pro-inflammatory (pro-inflam.) macrophage and microglia. **c-d**, UMAP annotated by pathology (anaplastic astrocytoma (a.astrocytoma), anaplastic glioma (a.glioma), diffuse intrinsic pontine glioma (DMG), group 3 (Gr3) and group 4 (Gr4) medulloblastoma (MB), posterior-fossa -type A (PFA) ependymoma type 1 and 2, posterior-fossa -type B (PFB) ependymoma, sonic-hedghog (SHH) medulloblastoma, Supratentorial-REL-associated protein (ST-RELA) ependymoma (EPN), ST-Yes1 associated transcriptional regulator (YAP) EPN) and development stage. **e-f** quantification of the absolute number (e) and proportion (f) of HSPCs across pathologies. **g**, Dotplot of mean expression and fraction of cells in group expressing cell type annotations genes across annotated cell types.

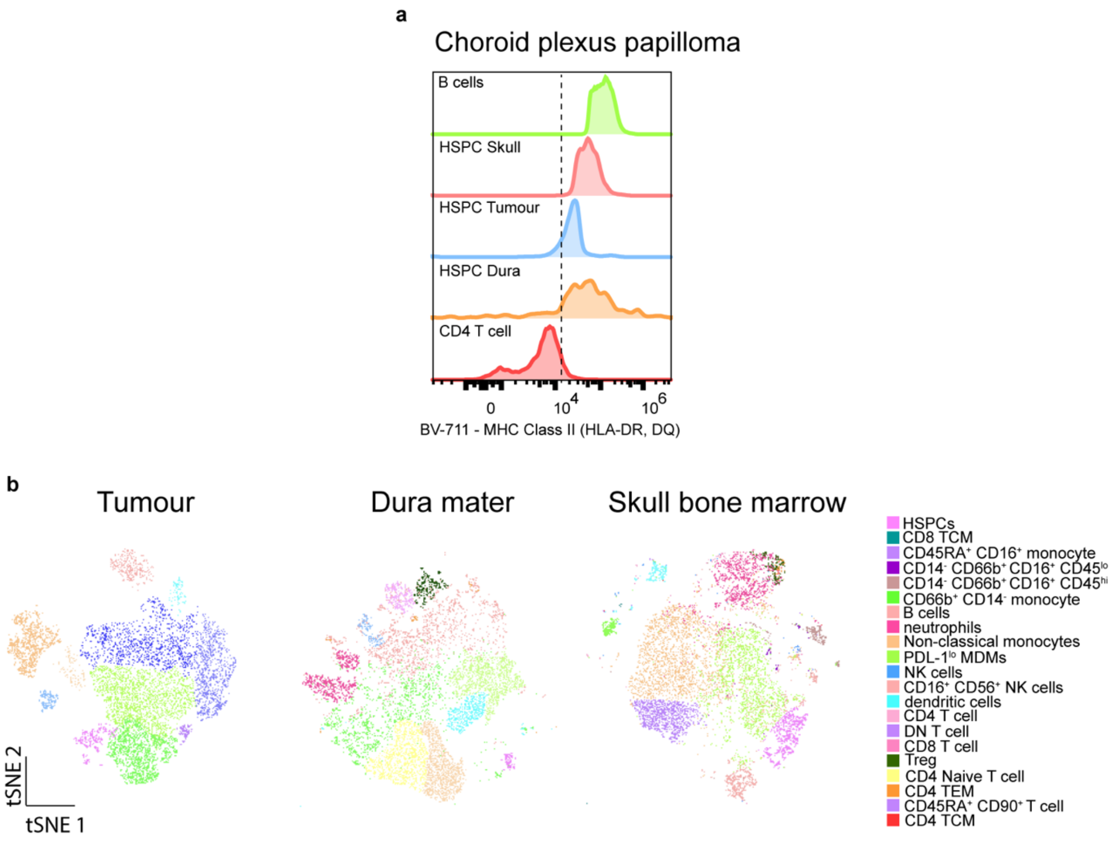

Supplementary Fig. 4 Human choroid plexus carcinoma neurosurgical CNS immune tissue reveals HLA-DR-expressing haematopoietic stem progenitor cells (HSPCs) in skull, dura and tumour parenchyma. a, Representative histogram showing HLA-DR expression levels in immune cell populations across tissues. **b**, Representative t-SNE of flow cytometry data from tumour parenchyma, dura mater and skull bone marrow with 3000 events concatenated per sample, manually gated; HSPCs, CD8 tissue central memory (TCM), CD45RA^{+} CD16^{+} monocytes, CD14^{-}CD66b^{+}CD16^{+}CD45^{lo}, CD14^{-}CD66b^{+}CD16^{+}CD45^{hi}, CD66b^{+}CD14^{-}, B cells, neutrophils, non-classical monocytes, PDL-1^{lo} monocye-derived macrophages (MDMs), natural killer (NK) cells CD16^{+}CD56^{+} NK cells, dendritic cells, CD4 T cells, double-negative (DN) T cells, CD8 T cell, regulatory T cell (Treg), CD4 Naïve T cell, CD4 tissue effector memory (TEM), CD45RA^{+} CD90^{+} T cell, CD4 TCM.

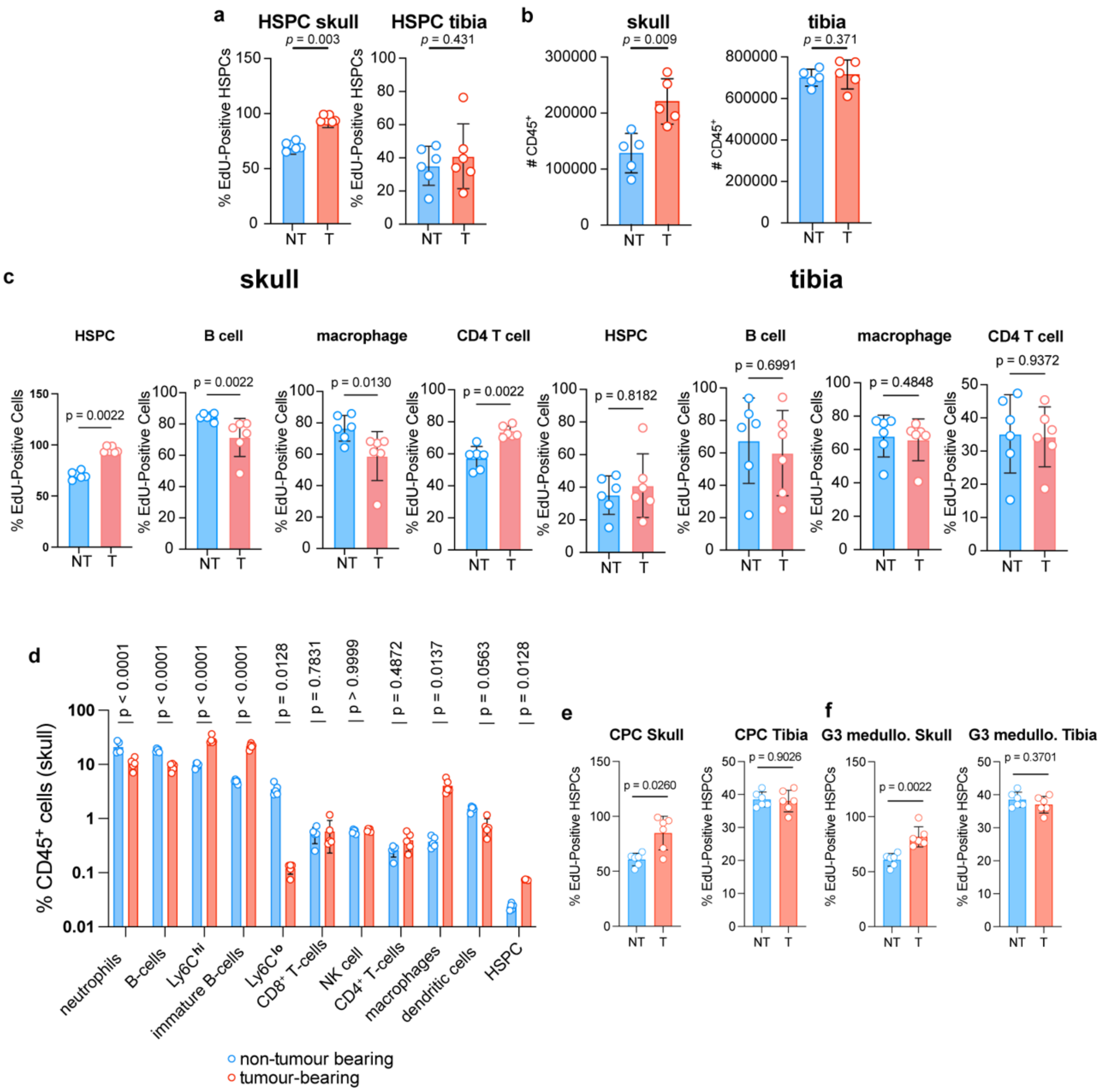

a
HSPC skull
p = 0.003
% EdU-Positive HSPCs
HSPC tibia
p = 0.431
NT
T
b
skull
p = 0.009
# CD45+
tibia
p = 0.371
c
skull
tibia
HSPC
B cell
macrophage
CD4 T cell
% EdU-Positive Cells
p = 0.0022
p = 0.0022
p = 0.0130
p = 0.0022
p = 0.8182
p = 0.6991
p = 0.4848
p = 0.9372
d
% CD45+ cells (skull)
p < 0.0001
p < 0.0001
p < 0.0001
p < 0.0001
p = 0.0128
p = 0.7831
p > 0.9999
p = 0.4872
p = 0.0137
p = 0.0563
p = 0.0128
neutrophils
B-cells
Ly6Chi
immature B-cells
Ly6Clo
CD8+ T-cells
NK cell
CD4+ T-cells
macrophages
dendritic cells
HSPC
non-tumour bearing
tumour-bearing
e
CPC Skull
p = 0.0260
CPC Tibia
p = 0.9026
f
G3 medullo. Skull
p = 0.0022
G3 medullo. Tibia
p = 0.3701
% EdU-Positive HSPCs

Supplementary Fig. 5 Local increased proliferation of skull bone marrow cells in $EP^{ZFTA\text{-}RELA}$**-bearing mice, which is absent in peripheral bone marrow niches. a,** Quantification of the percentage of 5-Ethynyl-2′-deoxyuridine (EdU)-positive flow cytometry profiled LSK^{+} HSPCs from the skull and tibia bone marrow of $EP^{ZFTA\text{-}RELA}$ -bearing and *and* $Nestin^{CreERT2}$, control bearing mice, n = 6 individual mice, average of 3 independent experiments, data are means ± s.e.m.; P values represent a two-sided Student's t-test. **b**, Number of $CD45^{+}$ cells in skull and tibia of $EP^{ZFTA\text{-}RELA}$-bearing and control bearing mice, quantified from **a.** **c,** Quantification of the percentage of EdU-positive immune cell subsets, n = 6 individual mice, average of 3 independent experiments, data are means ± s.e.m.; P values represent a two-sided Student's t-test. **d**, Quantification of the proportion of subsets of $CD45^{+}$ cells within the skull of $EP^{ZFTA\text{-}RELA}$ -bearing *and* $Nestin^{CreERT2}$ mice as measured by flow cytometry, (n=6/group, mean ± s.e.m, unpaired two-tailed Student's t-test). **e-f,** Quantification of the percentage of EdU-positive flow cytometry profiled LSK^{+} HSPCs from the skull and tibia bone marrow of orthotopic allotransplantation models of choroid plexus carcinoma and group 3 medulloblastoma, relative to sham control mice, n = 6 individual mice, average of 3 independent experiments, data are means ± s.e.m.; P values represent a two-sided Student's t-test.

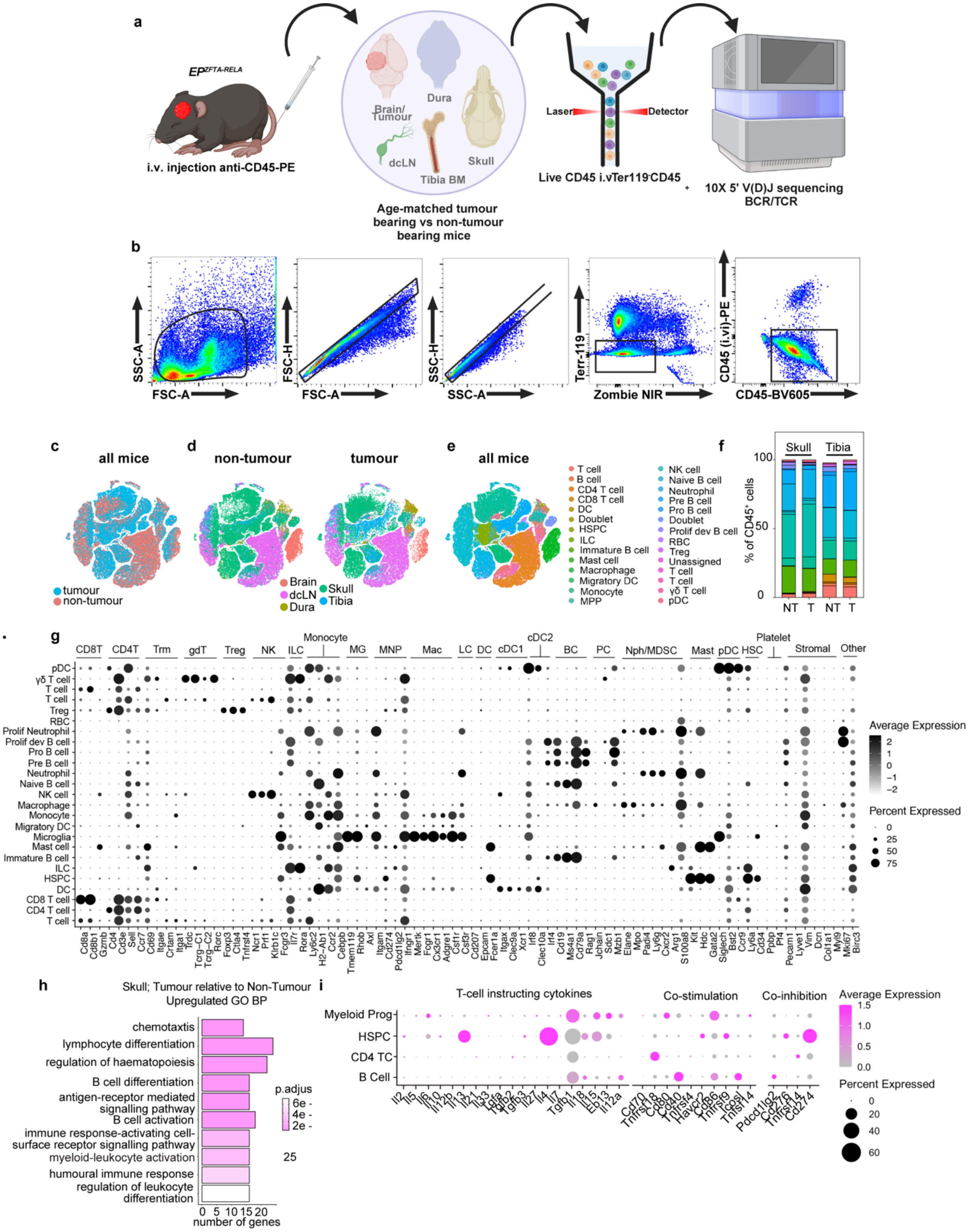
a
EP ZFTA-RELA
i.v. injection anti-CD45-PE
Brain/ Tumour
Dura
Skull
dcLN
Tibia BM
Age-matched tumour bearing vs non-tumour bearing mice
Laser
Detector
Live CD45 i.vTer119-CD45+
10X 5' V(D)J sequencing BCR/TCR
b
SSC-A
FSC-A
FSC-H
SSC-H
Terr-119
Zombie NIR
CD45 (i.v)-PE
CD45-BV605
c
all mice
tumour
non-tumour
d
non-tumour
tumour
Brain
dcLN
Dura
Skull
Tibia
e
all mice
T cell
B cell
CD4 T cell
CD8 T cell
DC
Doublet
HSPC
ILC
Immature B cell
Mast cell
Macrophage
Migratory DC
Monocyte
MPP
NK cell
Naive B cell
Neutrophil
Pre B cell
Pro B cell
Doublet
Prolif dev B cell
RBC
Treg
Unassigned
T cell
T cell
γδ T cell
pDC
f
Skull
Tibia
% of CD45+ cells
100
50
0
NT
T
NT
T
g
CD8T
CD4T
Trm
gdT
Treg
NK
ILC
Monocyte
MG
MNP
Mac
LC
DC
cDC1
cDC2
BC
PC
Nph/MDSC
Mast
pDC
HSC
Platelet
Stromal
Other
Average Expression
Percent Expressed
h
Skull; Tumour relative to Non-Tumour Upregulated GO BP
chemotaxtis
lymphocyte differentiation
regulation of haematopoiesis
B cell differentiation
antigen-receptor mediated signalling pathway
B cell activation
immune response-activating cell-surface receptor signalling pathway
myeloid-leukocyte activation
humoural immune response
regulation of leukocyte differentiation
p.adjus
number of genes
i
T-cell instructing cytokines
Co-stimulation
Co-inhibition
Myeloid Prog
HSPC
CD4 TC
B Cell
Average Expression
Percent Expressed

Supplementary Fig. 6 Experimental pipeline and cell type annotation of single-cell RNA-sequencing of mouse neuroimmune tissues in *$EP^{ZFTA\text{-}RELA}$*-**bearing and control bearing mice. a,** Schematic illustrating the methodology to isolate $CD45^{+}$ cells from the tissues listed in *$EP^{ZFTA\text{-}RELA}$*-bearing and control bearing mice, created in biorender.com. **b**, Representative gating strategy for the fluorescence-activated cell sorting (FACS) isolation of CD45 i.v- $Terr119^{-}$ Live $CD45^{+}$ cells. **c-e,** t-distributed stochastic neighbour embedding (t-SNE) visualizations of scRNA-seq from brain, deep-cervical lymph nodes (dcLN), dura, skull and tibial bone marrow from 4-week-old mice coloured by genotype, tissue – split by genotype and cell type. **f**, Stacked bar plot of the relative proportions of annotated scRNA-seq cell types within the skull and tibia bone marrow of *$EP^{ZFTA\text{-}RELA}$*-bearing and control bearing mice. **g**, Mean expression dot plot of marker genes used to define cell type annotations in single cell RNAseq data from the these tissues in *$EP^{ZFTA\text{-}RELA}$*-bearing and control bearing mice. **h**, Volcano plots of differentially expressed genes in neutrophils, monocytes, macrophages, and HSPCs within the skulls of tumour bearing relative to control bearing mice. Magenta dots represent upregulated transcripts, blue dots represent downregulated transcripts in skull populations compared to the tibia. y-axes represent adjusted $\log_2$ p value for cluster changes between tumour and control bearing mice. **i,** Dotplot of average and percentage expression of T cell instructing cytokines, co-inhibition and co-stimulation molecules across HSPC and myeloid progenitor cell clusters.

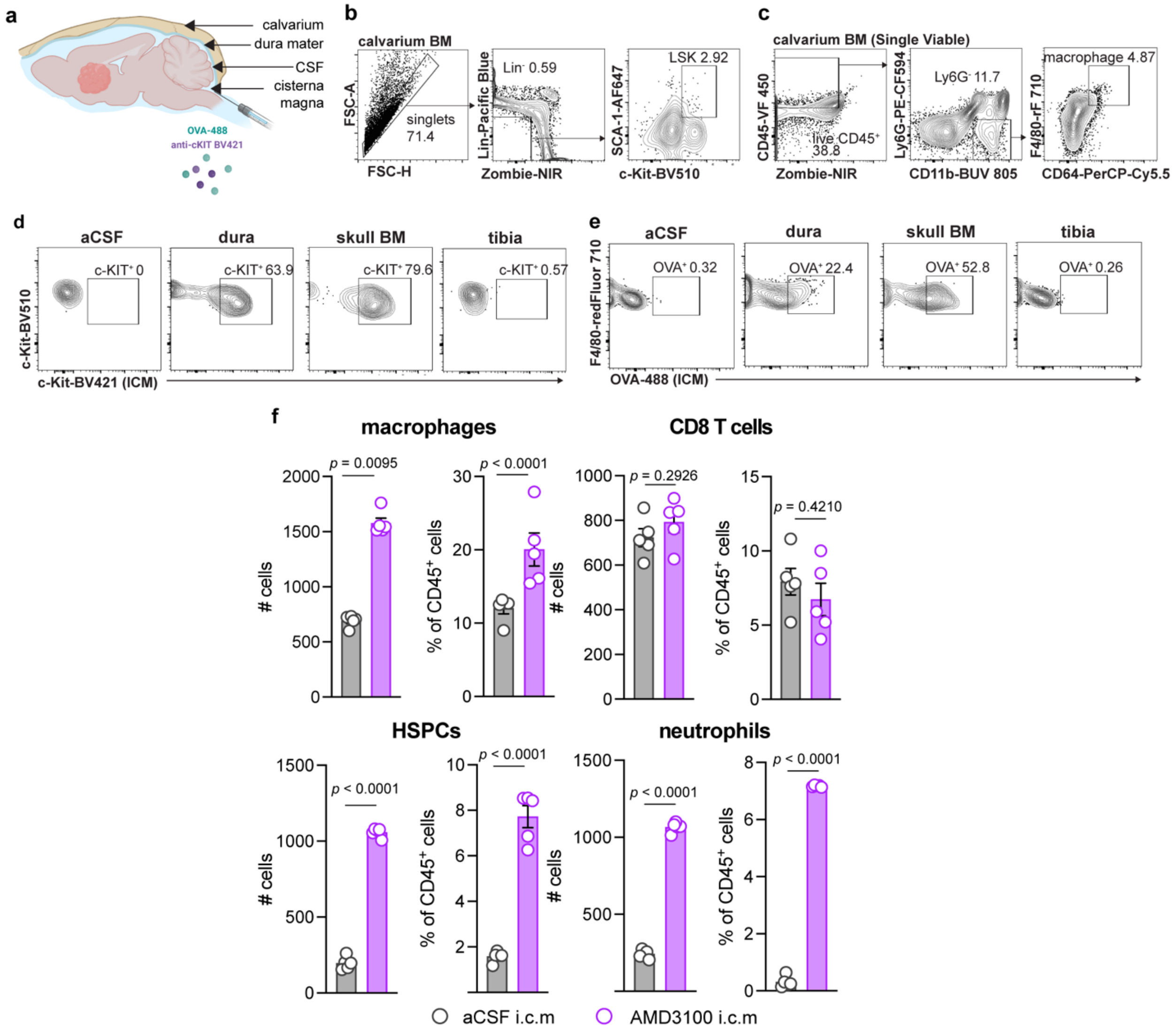

a
calvarium
dura mater
CSF
cisterna magna
OVA-488
anti-cKIT BV421
b
calvarium BM
singlets 71.4
Lin- 0.59
LSK 2.92
c
calvarium BM (Single Viable)
live CD45+ 38.8
Ly6G- 11.7
macrophage 4.87
d
aCSF
dura
skull BM
tibia
c-KIT+ 0
c-KIT+ 63.9
c-KIT+ 79.6
c-KIT+ 0.57
e
OVA+ 0.32
OVA+ 22.4
OVA+ 52.8
OVA+ 0.26
f
macrophages
CD8 T cells
HSPCs
neutrophils
p = 0.0095
p < 0.0001
p = 0.2926
p = 0.4210
# cells
% of CD45+ cells
aCSF i.c.m
AMD3100 i.c.m

Supplementary Fig. 7 CSF access and modulate skull bone marrow niche in EP$^{ZFTA\text{-}RELA}$**-bearing mice.** **a**, Experimental design of intracisterna magna (ICM) injections of anti-c-Kit BV421 and OVA–488 in tumor and non-tumor-bearing mice (n=5 per group); image created with Biorender.com. **b**, Gating strategy to quantify dual-positive c-Kit$^+$ cells, summarized in (f). **c**, Gating strategy to quantify OVA-488$^+$ macrophages in dura, tibia, and skull bone marrow. **d-e**, Representative flow cytometry plots of dual-positive c-Kit cells (**d**) and OVA-positive F4/80$^+$ macrophages (**e**) across experimental conditions. **f**, Quantification of the proportion and number of intratumoral macrophages, CD8 T cells, haematopoietic stem progenitor cells (HSPCs) and neutrophils following intracisternal magna injection of AMD3100 (2 mg/kg, 6 h) or artificial CSF (aCSF) treatment (n=5/group, mean±s.e.m, unpaired two-tailed Student's t-test) relative to aCSF).

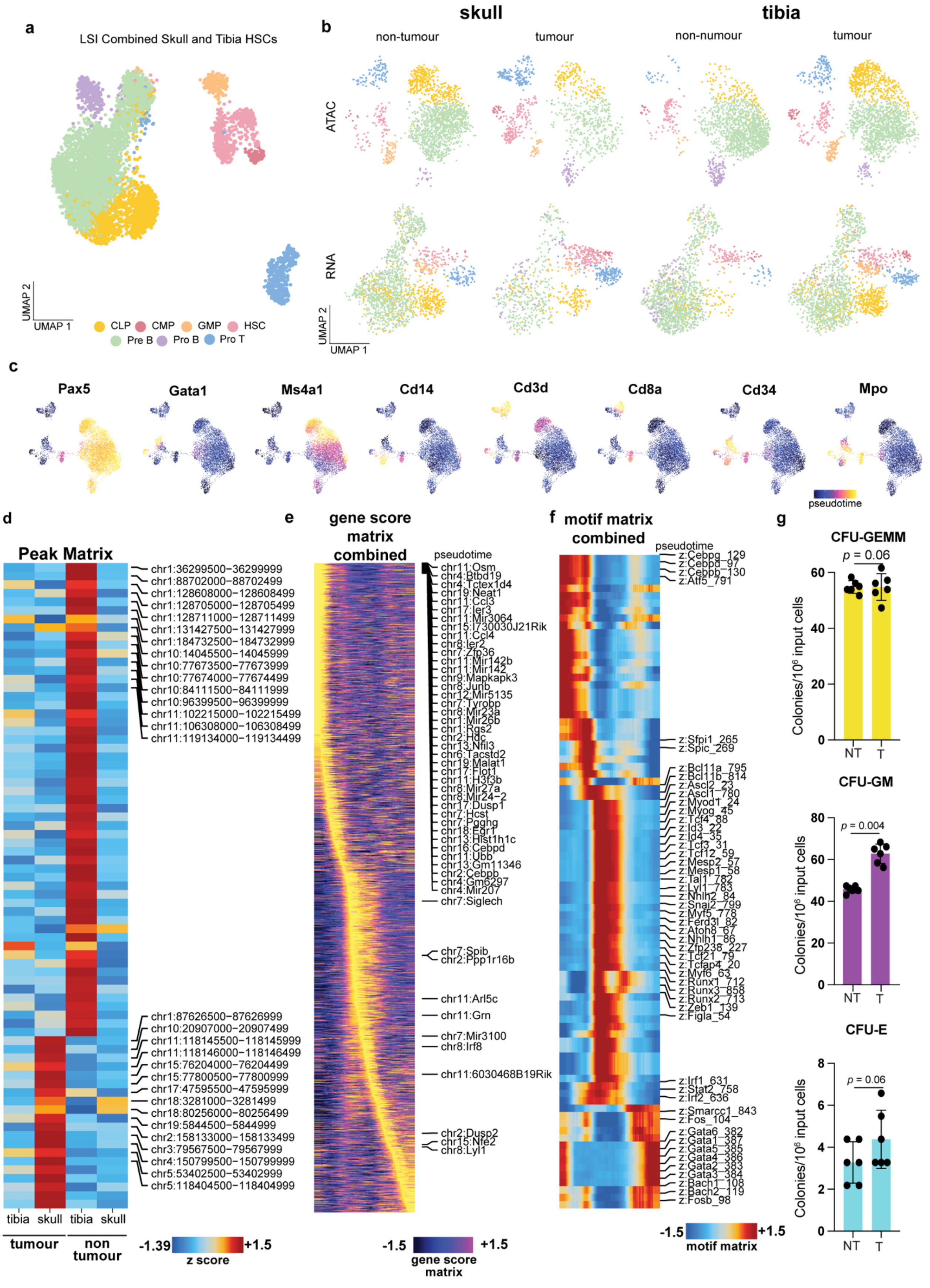
a
LSI Combined Skull and Tibia HSCs
UMAP 2
UMAP 1
CLP
CMP
GMP
HSC
Pre B
Pro B
Pro T
b
skull
tibia
non-tumour
tumour
non-numour
tumour
ATAC
RNA
UMAP 2
UMAP 1
c
Pax5
Gata1
Ms4a1
Cd14
Cd3d
Cd8a
Cd34
Mpo
pseudotime
d
Peak Matrix
chr1:36299500-36299999
chr1:88702000-88702499
chr1:128608000-128608499
chr1:128705000-128705499
chr1:128711000-128711499
chr1:131427500-131427999
chr1:184732500-184732999
chr10:14045500-14045999
chr10:77673500-77673999
chr10:77674000-77674499
chr10:84111500-84111999
chr10:96399500-96399999
chr11:102215000-102215499
chr11:106308000-106308499
chr11:119134000-119134499
chr1:87626500-87626999
chr10:20907000-20907499
chr11:118145500-118145999
chr11:118146000-118146499
chr15:76204000-76204499
chr15:77800500-77800999
chr17:47595500-47595999
chr18:3281000-3281499
chr18:80256000-80256499
chr19:5844500-5844999
chr2:158133000-158133499
chr3:79567500-79567999
chr4:150799500-150799999
chr5:53402500-53402999
chr5:118404500-118404999
tibia
skull
tibia
skull
tumour
non tumour
-1.39
+1.5
z score
e
gene score matrix combined
pseudotime
chr11:Osm
chr4:Btbd19
chr4:Tctex1d4
chr19:Neat1
chr11:Ccl3
chr17:Ier3
chr11:Mir3064
chr15:I730030J21Rik
chr11:Ccl4
chr8:Ier2
chr7:Zfp36
chr11:Mir142b
chr11:Mir142
chr9:Mapkapk3
chr8:Junb
chr12:Mir5135
chr7:Tyrobp
chr8:Mir23a
chr1:Mir26b
chr1:Rgs2
chr2:Hdc
chr13:Nfil3
chr6:Tacstd2
chr19:Malat1
chr17:Flot1
chr11:H3f3b
chr8:Mir27a
chr8:Mir24-2
chr17:Dusp1
chr7:Hcst
chr7:Pgghg
chr18:Egr1
chr13:Hist1h1c
chr16:Cebpd
chr11:Ubb
chr13:Gm11346
chr2:Cebpb
chr4:Gm6297
chr4:Mir207
chr7:Siglech
chr7:Spib
chr2:Ppp1r16b
chr11:Arl5c
chr11:Grn
chr7:Mir3100
chr8:Irf8
chr11:6030468B19Rik
chr2:Dusp2
chr15:Nfe2
chr8:Lyl1
-1.5
+1.5
gene score matrix
f
motif matrix combined
pseudotime
z:Cebpg_129
z:Cebpd_97
z:Cebpb_130
z:Atf5_791
z:Sfpi1_265
z:Spic_269
z:Bcl11a_795
z:Bcl11b_814
z:Ascl2_23
z:Ascl1_780
z:Myod1_24
z:Myog_45
z:Tcf4_88
z:Id3_22
z:Id4_35
z:Tcf3_31
z:Tcf12_59
z:Mesp2_57
z:Mesp1_58
z:Tal1_782
z:Lyl1_783
z:Nhlh2_84
z:Snai2_799
z:Myf5_778
z:Ferd3l_82
z:Atoh8_67
z:Nhlh1_86
z:Zfp238_227
z:Tcf21_79
z:Tcfap4_20
z:Myf6_63
z:Runx1_712
z:Runx3_858
z:Runx2_713
z:Zeb1_139
z:Figla_54
z:Irf1_631
z:Stat2_758
z:Irf2_636
z:Smarcc1_843
z:Fos_104
z:Gata6_382
z:Gata1_387
z:Gata5_385
z:Gata4_386
z:Gata2_383
z:Gata3_384
z:Bach1_108
z:Bach2_119
z:Fosb_98
-1.5
+1.5
motif matrix
g
CFU-GEMM
p = 0.06
Colonies/10^6 input cells
NT
T
CFU-GM
p = 0.004
Colonies/10^6 input cells
NT
T
CFU-E
p = 0.06
Colonies/10^6 input cells
NT
T

Supplementary Fig. 8 Combined analysis of chromatin accessibility and gene expression in HSCs from skull and tibia of EP$^{ZFTA\text{-}RELA}$**-bearing and control-bearing mice.** **a**, Uniform manifold approximation and projection (UMAP) visualization of combined single nucleus assay for transposase-accessible chromatin using sequencing (snATAC-seq) and RNA data using latent semantic indexing (LSI) from fluorescence-activated cell sorting (FACS)-isolated CD34^{+} LSK^{+} (haematopoietic stem cell (HSC), showing distinct clustering of hematopoietic subpopulations, common myeloid progenitor (CMP), granulocyte monocyte progenitor (GMP), Pro B, and Pro T) across skull and tibia samples. **b**, UMAP plots of snATAC (top) and RNA (bottom) data, stratified by tumour and control conditions, highlighting the distribution of hematopoietic subpopulations in skull and tibia. **c**, Feature plots of key lineage-specific genes (Pax5, Gata1, Ms4a1, Cd14, Cd3d, Cd8a, Cd34, and Mpo), illustrating expression dynamics across hematopoietic differentiation trajectories. **d**, Heatmap of the peak matrix, showing Z-scores for differentially accessible chromatin regions across tumour and control skull and tibia samples. **e**, Heatmap of the combined gene score matrix across pseudo time, highlighting dynamically regulated genes (Spib, Siglech, Ppp1r16b, Arl5c, and Dusp2) associated with differentiation. **f**, Heatmap of the motif matrix, showing activity scores of transcription factor motifs (Cebpb, Cebpg, Zbtb16, Fosb, Bach1, and Smarcc1) across pseudotime, with increased motif activity in EP$^{ZFTA\text{-}RELA}$-bearing samples indicative of enhanced regulatory engagement. **g**, Quantification of the number and types of colonies (colony forming unit (CFU)-granulocytes/erythroids/macrophages/megakaryocytes (CFU-GEMM), colony-forming unit-granulocytes/macrophages (CFU-GM) and colony-forming unit-erythroid (CFU-E) in colony forming cell assays derived from skull bone marrow from EP$^{ZFTA\text{-}RELA}$-bearing and control bearing mice, (n=6/group, mean±s.e.m, unpaired two-tailed Student's t-test).

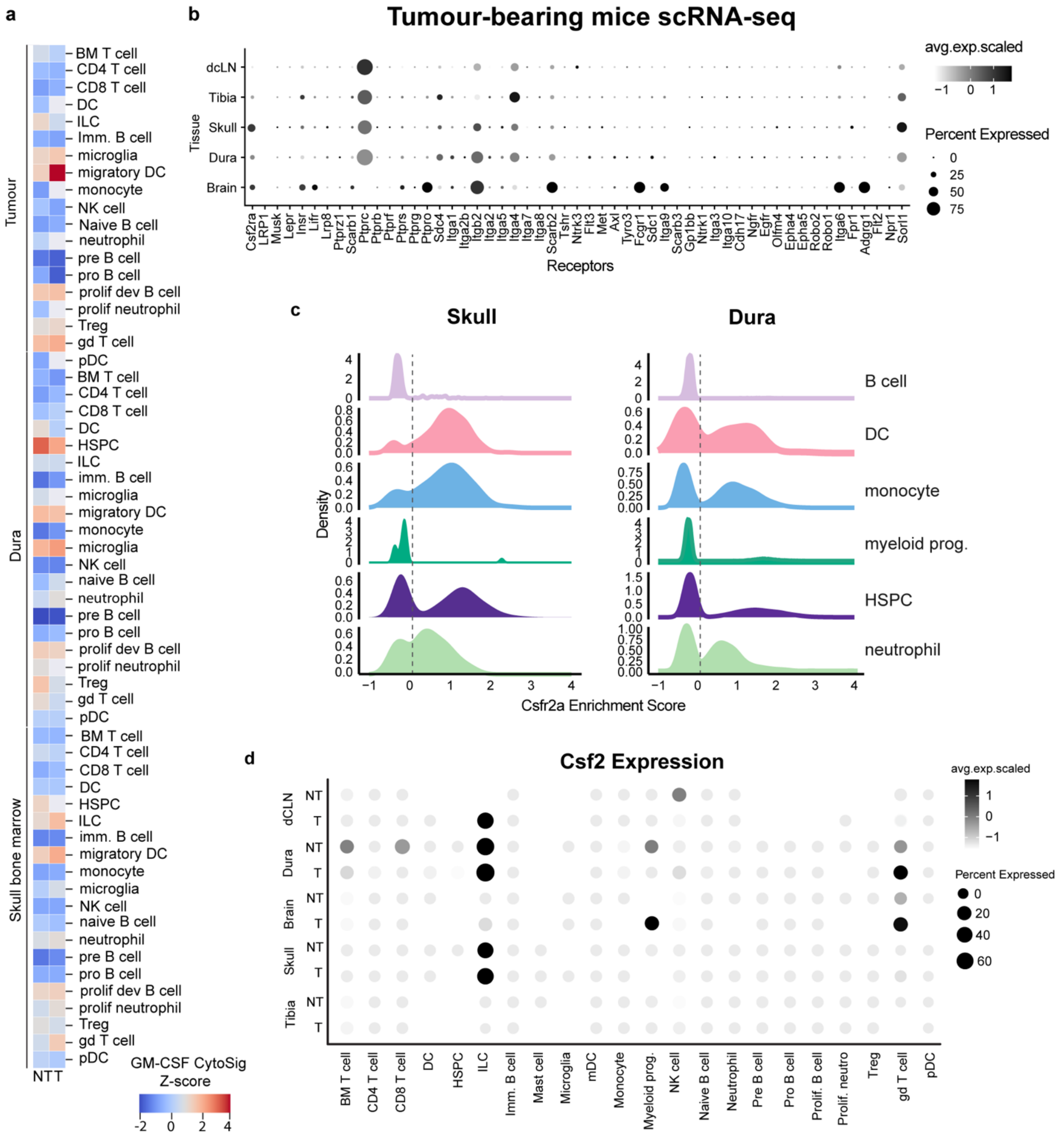

a
b
Tumour-bearing mice scRNA-seq
c
Skull
Dura
Csfr2a Enrichment Score
d
Csf2 Expression
GM-CSF CytoSig Z-score

Supplementary Fig. 9 Single-cell RNA-sequencing highlights CSFR2A expression on skull and dura in *EP*$^{ZFTA\text{-}RELA}$-bearing mice. **a**, Heatmap depicting GM-CSF (Csf2)-associated cytokine enrichment scores generated using CytoSig for annotated cell types across skull, dura, and brain from control and EP$^{ZFTA\text{-}RELA}$ -bearing mice. **b**, Dot plot of receptor expression in skull bone marrow cells of *EP*$^{ZFTA\text{-}RELA}$-bearing mice, scaled by gene expression and percentage of cells expressing the gene. **c**, Smooth density plots of CSFR2a enrichment scores across selected immune cell types in the Skull and Dura tissues. **d**, Dot plot of CSF2 expression in across cells and tissues of *EP*$^{ZFTA\text{-}RELA}$-bearing and control bearing mice, scaled by gene expression and percentage of cells expressing the gene.

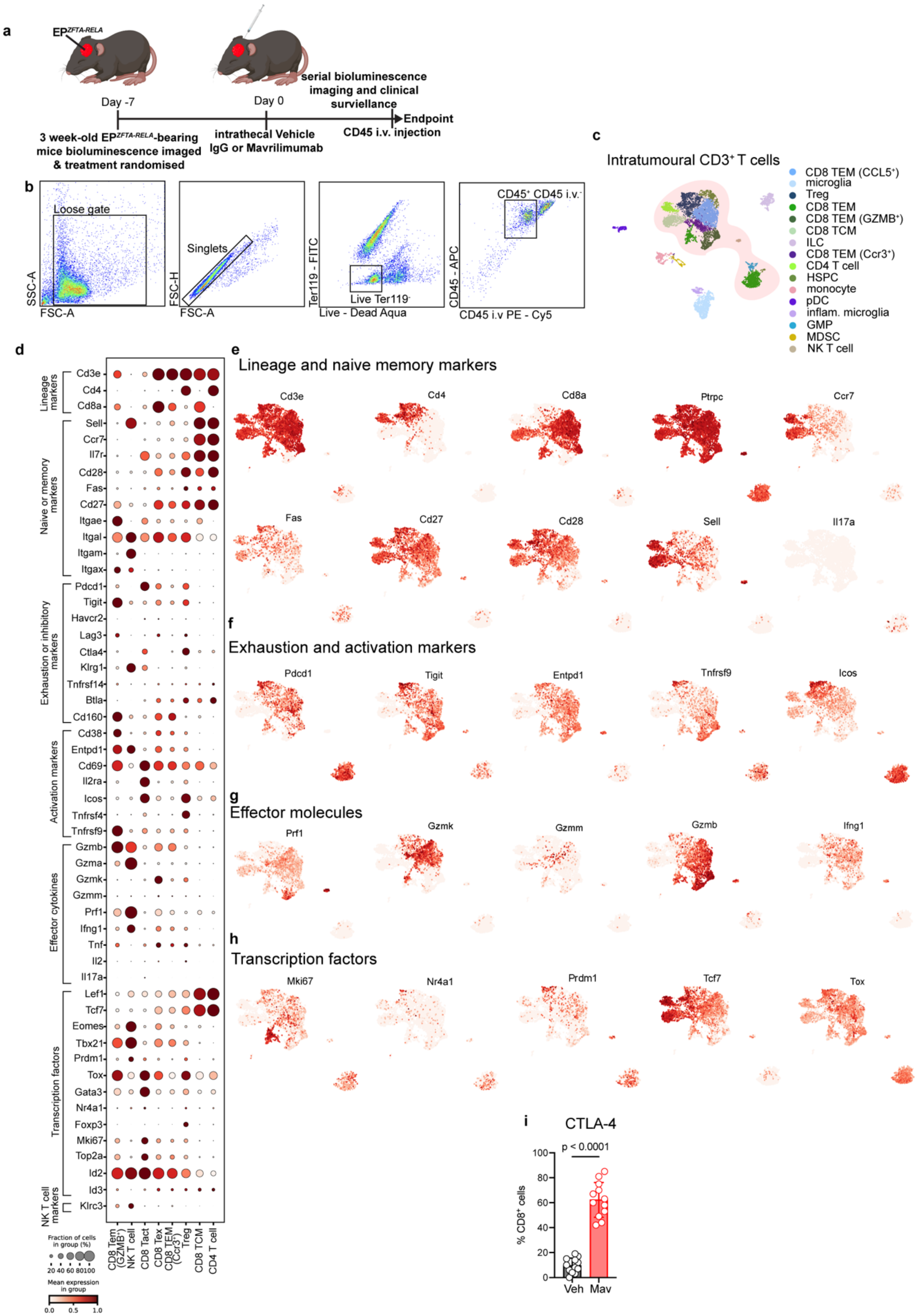
a
EP^ZFTA-RELA
Day -7
Day 0
serial bioluminescence imaging and clinical surviellance
Endpoint
CD45 i.v. injection
3 week-old EP^ZFTA-RELA-bearing mice bioluminescence imaged & treatment randomised
intrathecal Vehicle IgG or Mavrilimumab
b
Loose gate
SSC-A
FSC-A
Singlets
FSC-H
FSC-A
Ter119 - FITC
Live Ter119-
Live - Dead Aqua
CD45+ CD45 i.v.-
CD45 - APC
CD45 i.v PE - Cy5
c
Intratumoural CD3+ T cells
CD8 TEM (CCL5+)
microglia
Treg
CD8 TEM
CD8 TEM (GZMB+)
CD8 TCM
ILC
CD8 TEM (Ccr3+)
CD4 T cell
HSPC
monocyte
pDC
inflam. microglia
GMP
MDSC
NK T cell
d
Lineage markers
Cd3e
Cd4
Cd8a
Naive or memory markers
Sell
Ccr7
Il7r
Cd28
Fas
Cd27
Itgae
Itgal
Itgam
Itgax
Exhaustion or inhibitory markers
Pdcd1
Tigit
Havcr2
Lag3
Ctla4
Klrg1
Tnfrsf14
Btla
Cd160
Activation markers
Cd38
Entpd1
Cd69
Il2ra
Icos
Tnfrsf4
Tnfrsf9
Effector cytokines
Gzmb
Gzma
Gzmk
Gzmm
Prf1
Ifng1
Tnf
Il2
Il17a
Transcription factors
Lef1
Tcf7
Eomes
Tbx21
Prdm1
Tox
Gata3
Nr4a1
Foxp3
Mki67
Top2a
Id2
Id3
NK T cell markers
Klrc3
CD8 Tem (GZMB+)
NK T cell
CD8 Tact
CD8 Tex
CD8 TEM (Ccr3+)
Treg
CD8 TCM
CD4 T cell
Fraction of cells in group (%)
20 40 60 80 100
Mean expression in group
0.0 0.5 1.0
e
Lineage and naive memory markers
Cd3e
Cd4
Cd8a
Ptprc
Ccr7
Fas
Cd27
Cd28
Sell
Il17a
f
Exhaustion and activation markers
Pdcd1
Tigit
Entpd1
Tnfrsf9
Icos
g
Effector molecules
Prf1
Gzmk
Gzmm
Gzmb
Ifng1
h
Transcription factors
Mki67
Nr4a1
Prdm1
Tcf7
Tox
i
CTLA-4
p < 0.0001
% CD8+ cells
0
20
40
60
80
100
Veh
Mav

Supplementary Fig. 10. Increased T cell infiltrate in single-cell RNAseq of intratumoural immune compartment following treatment with mavrilimumab in EP$^{ZFTA-RELA}$ mice. a, Experimental design for the treatment of 3 week-old EP$^{ZFTA-RELA}$-bearing mice with a single intracisternal magna (ICM) injection of 10 uL of IgG isotype control (5 mg/kg, n = 6 per group or mavrililumab (5 mg/kg, n = 6 per group) created with biorender.com. **b**, Representative flow cytometry plots for the enrichment of extravascular CD45^{+} cells. **c**, Uniform manifold approximation and projection visualisation of CD45^{+} cells in integrated vehicle and mavrilimumab single-cell RNAseq datasets. **d**, Dotplot of expression of T cell lineage and phenotype markers across annotated cell types. **e-g**, Featureplot of key phenotypic marker expression levels including **(e)** lineage and memory, **(f)** exhaustion and activation, **(g)** effector molecule, and **(h)** transcription factors genes. **i**, Flow cytometry quantification of the proportion of CTLA-4^{+} CD8 T cells in tumour parenchyma of treated mice at recurrence, n = 12 mice per group.
