# Supplementary material for: Childhood brain tumors instruct cranial hematopoiesis and immunotolerance
Source: Nat Genet. 2026 Feb 3;58(2):317–28. doi: 10.1038/s41588-025-02499-2 (PMC12900635; doi:10.1038/s41588-025-02499-2)
Supplement: Supplementary file 2 — Reporting Summary [file 41588_2025_2499_MOESM2_ESM.pdf]

nature portfolio

Corresponding author(s): Richard J. Gilbertson and Elizabeth Cooper

Last updated by author(s): 1/12/2025

Reporting Summary

Nature Portfolio wishes to improve the reproducibility of the work that we publish. This form provides structure for consistency and transparency in reporting. For further information on Nature Portfolio policies, see our Editorial Policies and the Editorial Policy Checklist.

Statistics

For all statistical analyses, confirm that the following items are present in the figure legend, table legend, main text, or Methods section.

n/a	Confirmed	
☐	☒	The exact sample size (*n*) for each experimental group/condition, given as a discrete number and unit of measurement
☐	☒	A statement on whether measurements were taken from distinct samples or whether the same sample was measured repeatedly
☐	☒	The statistical test(s) used AND whether they are one- or two-sided<br>*Only common tests should be described solely by name; describe more complex techniques in the Methods section.*
☒	☐	A description of all covariates tested
☐	☒	A description of any assumptions or corrections, such as tests of normality and adjustment for multiple comparisons
☐	☒	A full description of the statistical parameters including central tendency (e.g. means) or other basic estimates (e.g. regression coefficient) AND variation (e.g. standard deviation) or associated estimates of uncertainty (e.g. confidence intervals)
☐	☒	For null hypothesis testing, the test statistic (e.g. *F*, *t*, *r*) with confidence intervals, effect sizes, degrees of freedom and *P* value noted<br>*Give P values as exact values whenever suitable.*
☒	☐	For Bayesian analysis, information on the choice of priors and Markov chain Monte Carlo settings
☒	☐	For hierarchical and complex designs, identification of the appropriate level for tests and full reporting of outcomes
☒	☐	Estimates of effect sizes (e.g. Cohen's *d*, Pearson's *r*), indicating how they were calculated

Our web collection on statistics for biologists contains articles on many of the points above.

Software and code

Policy information about availability of computer code

Data collection	SpectroFlo v 2.2.0.3 (Cytek)<br>Leica Stellaris 8 - MP<br>Luminex FLEXMAP 3D
Data analysis	Detailed analysis methods can be found in the methods section of the manuscript<br>Packages and software used include: Cell Ranger ARC Suite (v.2.0.0), MACS2(v2.2.7), Seurat v4.3.0, Seuratv5.0.1, SCTransform v2 (v0.4.1), limma (v3.58.1), chromVAR (v1.16.0), ArchR (v1.0.3), scanpy (v1.11.3), Dandelion (v0.5.5)<br>FIJI image processing software (NIH) - v 2.14.0/1.54f<br>Prism v 10.2.0 (GraphPad Software, Inc)<br>FastQC v 0.11.5 - R v 3.5.0<br>RStudio v 1.4.1717<br>Bioconductor DESeq2 v3.5<br>FlowJo software v 10 (BD Biosciences)

For manuscripts utilizing custom algorithms or software that are central to the research but not yet described in published literature, software must be made available to editors and reviewers. We strongly encourage code deposition in a community repository (e.g. GitHub). See the Nature Portfolio guidelines for submitting code & software for further information.

Data

Policy information about availability of data

All manuscripts must include a data availability statement. This statement should provide the following information, where applicable:

- Accession codes, unique identifiers, or web links for publicly available datasets
- A description of any restrictions on data availability
- For clinical datasets or third party data, please ensure that the statement adheres to our policy

The accession number for the Fastq files and quantified gene counts for single-cell sequencing reported in this paper is GEO: GSE28237, GSE82459, GSE300889 and GSE300890. All data are available in the main text or the Supplementary Information files. Data were also sourced from the following published accessions: NEMO; dat-oii74w and dat-3ah9h9x, GEO; GSE141460, GSE126025, GSE156053, GSE226961 and GSE231860. The mass spectrometry proteomics data have been deposited to the ProteomeXchange Consortium via the PRIDE partner repository with the dataset identifier PXD058239.

Research involving human participants, their data, or biological material

Policy information about studies with human participants or human data. See also policy information about sex, gender (identity/presentation), and sexual orientation and race, ethnicity and racism.

Reporting on sex and gender	Sex and gender were not used in any scenario as criteria for sample collection. Sex of 2nd trimester de-identified samples were determined based on sex-specific gene expression patterns as determined in previously published datasets. Sex information from all other deidentified samples were provided by the relevant brain and tissue banks. Both male and female samples were treated equally.
Reporting on race, ethnicity, or other socially relevant groupings	No race, ethnicity or socially relevant groupings were performed in this study
Population characteristics	We collected neurosurgical material from a single 6 mo old M, diagnosed with Atypical choroid plexus papilloma (CNS Who Grade 2), no prior treatments
Recruitment	De-identified tissue samples were collected with previous patient consent in strict observance of the legal and institutional ethical regulations. This was performed by the clinic and the inclusion criteria was <18 years of age, primary primary tumour diagnosis. Because we have no demographic information about our sample or the patient population we cannot comment on how any bias may or may not have been present.
Ethics oversight	Ethical approval was given through Cambridge central Research Ethics Committee (23/EE/0241), administrered through Cambridge University Hospitals NHS Foundaation Trust. All subjects provided informed written consent

Note that full information on the approval of the study protocol must also be provided in the manuscript.

Field-specific reporting

Please select the one below that is the best fit for your research. If you are not sure, read the appropriate sections before making your selection.

☒ Life sciences ☐ Behavioural & social sciences ☐ Ecological, evolutionary & environmental sciences

For a reference copy of the document with all sections, see nature.com/documents/nr-reporting-summary-flat.pdf

Life sciences study design

All studies must disclose on these points even when the disclosure is negative.

Sample size	Sample size: Statistical methods were not used to recalculate or predetermine study sizes but were based on similar experiments previously published (Cugurra et al., Science (2021); Rustenhoven et al., Cell (2021); Mazzitelli et al. (2022)).
Data exclusions	No data were excluded for analysis
Replication	All experiments were replicated in at least two independent experiments for a total of at least 3 mice per group, and all replication was successful. All representative images are representative of the same experiment performed in at least 3 animals. For in vitro experiments, cells from the same animal were resuspended and plated with treatments and controls in adjacent wells. Single cell RNA sequencing finding have been validated by microscopy and/or flow cytometry as stated in the text. All attempts at replication were successful
Randomization	For all experiments, animals from different cages were randomly assigned to different experimental groups. Because all variables were controlled for, no covariates were present. For in vitro studies, cells from the same animal were harvested and resuspended. Treatments and controls were plated adjacent to one another.
Blinding	For all experiments, the researchers were blinded, where possible, for at least one of the independent experiments. These experiments included dural wholemount analysis, and anti-GM-CSF treamtments.

Reporting for specific materials, systems and methods

We require information from authors about some types of materials, experimental systems and methods used in many studies. Here, indicate whether each material, system or method listed is relevant to your study. If you are not sure if a list item applies to your research, read the appropriate section before selecting a response.

Materials & experimental systems

n/a	Involved in the study
☐	☒ Antibodies
☒	☐ Eukaryotic cell lines
☒	☐ Palaeontology and archaeology
☐	☒ Animals and other organisms
☐	☒ Clinical data
☒	☐ Dual use research of concern
☒	☐ Plants

Methods

n/a	Involved in the study
☒	☐ ChIP-seq
☐	☒ Flow cytometry
☒	☐ MRI-based neuroimaging

Antibodies

Antibodies used

CD11c Armenian Hamster APC N418 117310 BioLegend 1:100CD11c Armenian Hamster BV786 HL3 563735 BD Biosciences 1:250
CD161 (NK1.1) Mouse BV480 PK136 108736 BioLegend 1:500 Ly-6C Rat APC-Cy7 HK1.4 128026 BioLegend 1:200
Ly-6G Rat PE-CF594 1A8 562700 BD Biosciences 1:400
CD163 Rat BUV661 Mac-2-158 156704 BioLegend 1:200
CD45R (B220) Rat BV750 RA3-6B2 747469 BD Biosciences 1:500
CD45R (B220) Rat eFluor 615 RA3-6B2 42-0452-82 Invitrogen 1:200
MHC Class II (I-Ad) Rat Pe-Cy7 M5/114.14.2 60-5321 Cytek Biosciences 1:1000
MHC Class II (I-AI/I-E) Rat BV650 M5/114.14.2 107641 BioLegend 1:1000
CD45 Rat violetFluor450 30-F11 75-0451-U100 Tonbo Biosciences 1:500
CX3CR1 Rat BB515 Z80-50 567809 BD Biosciences 1:400
CD11b Rat BUV805 M1/70 566416 BD Biosciences 1:1200
CD206 Rat BV711 C068C2 141727 BioLegend 1:200
CD3 Rat Spark NIR 685 17A2 100262 BioLegend
CD172a Rat RY586 P84 753227 BD Biosciences 1:100
CD8 Rat PE-Cy7 2.43 60-1886-U100 Tonbo Biosciences 1:400
F4-80 Rat redfluor710 BM8.1 80-4801-U100 Tonbo Biosciences 1:100
CD127 (IL-7Ra) Rat BV786 SB/199 563748 BD Biosciences 1:100
CD49b Rat eFluor 506 Dx5 69-5971-82 Invitrogen 1:200
CD47 Rat BB700 miap301 742181 BD Biosciences 1:100
CD4 Rat BV421 GK1.5 100443 BioLegend 1:5000
CD4 Rat PE GK1.5 100408 BioLegend 1:200
CD86 Rat SuperBright 436 B7-2 62-0862-82 Invitrogen 1:200
P2RY12 Rat APC-Fire 810 S16007D 848013 BioLegend 1:200
CD69 Armenian Hamster PerCP H1.2F3 104520 BD Biosciences 1:100
CD62L (L-Selectin) Rat APC-Cy7 MEL-14 25-0621-U100 Tonbo Biosciences 1:800
CD44 Rat AlexaFluor 700 IM7 560567 BD Biosciences 1:200
TCRγδ Armenian Hamster BUV737 GL3 748991 BioLegend 1:200
CD25 Rat BV711 PC61 102049 BioLegend 1:400
CD366 (TIM3) Rat BV605 RMT3-23 119721 119721 1:100
CD223 (LAG-3) Rat BV650 C9B7W 740560 BD Biosciences 1:100
CD19 Rat Super Bright 780 1D3 78-0193-82 Invitrogen 1:400
CD11a Rat BUV 496 2D7 741056 BD Biosciences 1:200
CD49d Rat PerCP-Cyanine 5.5 R1-2 65-0492-U100 Tonbo Biosciences 1:400
MHC Class I (HLA-G) Rat BUV 563 M1/42 749703 BD Biosciences 1:400
CD152 (CTLA-4) Armenian Hamster PE UC10-4F10-11 553720 BD Biosciences 1:100
CD274 Rat BUV615 MIH5 752339 BD Biosciences 1:200
Lineage Rat Pacific Blue 17A2; RB6-8C5; RA3-6B2; Ter-119; M1/70; 133310 BioLegend 1:500
CD117 Rat BV421 2B8 105828 BioLegend 1:500
CD117 Rat BV605 2B8 105847 BioLegend 1:300
Sca-1 Rat Super Bright 660 D7 64-5981-82 Cytek Biosciences 1:500
Sca-1 Rat AlexaFluor 647 D7 108118 BioLegend 1:200
FOXP3 Rat AlexaFluor 647 MF23 560401 BioLegend 1:200
GATA3 Mouse AlexaFluor 488 16E10A23 653807 BioLegend 1:200
Ki67 Mouse BV605 B56 567122 BD Biosciences 1:100
RORγt Mouse BV650 Q31-378 564722 BD Biosciences 1:200
T-bet Mouse PerCP-Cy5.5 4B10 644806 BioLegend 1:50
TCRVb Armenian Hamster BUV805 H57-597 748405 BD Biosciences 1:200
TCRvB11 Rat Pe-Cy7 KT-11 125916 BioLegend 1:200
CD64 Mouse BV421 X54-5/7.1 139309 BioLegend 1:200
CD115 Rat BUV737 AFS98 750948 BD Biosciences 1:200

XCR1 Mouse BV510 ZET 148220 BioLegend 1:200
TCRγδ Human REA633 REA633 130117111 MiltenyiBiotec 1:50
RFP Rabbit FITC - ab34764 Abcam 1:200
CD150 Rat BV650 TC15-12F12.2 115932 BioLegend 1:400
CD41 Rat FITC MWReg30 133904 BioLegend 1:250
CD16/32 Rat PeDazzle S17011E 156616 BioLegend 1:200
CD105 Rat RB780 MJ7/18 755709 BD Biosciences 1:100
CD48 Rat BB700 HM48-1 742119 BD Biosciences 1:200
CD34 Rat eFluor450 Ram34 48-0341-82 Invitrogen 1:200
CD184 Rat BUV805 2B11/CXCR4 741979 BD Biosciences 1:800
CD201 Rat PerCP-eFluor 710 eBio1560 (1560) 46-2012-80 Invitrogen 1:400
CD43 Rat PerCP 1B11 121222 BioLegend 1:200

Human
BD Horizon™ BUV737 Mouse Anti-Human CD16 BUV737 564434 BD Biosciences 1:200
BD OptiBuild™ BUV496 Mouse Anti-Human CD44 BUV496 750519 BD Biosciences 1:500
Alexa Fluor® 700 anti-human CD45 Antibody AF700 304024 Biolegend 1:500
PE/Cyanine7 anti-human CD66b Antibody - G10F5 Pe Cy7 305116 Biolegend 1:500
PerCP anti-human CD14 Antibody PerCP 325632 Biolegend 1:500
Brilliant Violet 510™ anti-human CD49d Antibody BV510 304318 Biolegend 1:500
Brilliant Violet 711™ anti-human HLA-DR Antibody BV711 307644 Biolegend 1:500
PE/Dazzle™ 594 anti-human CD56 (NCAM) Antibody PeDazzle 318348 Biolegend 1:500
Brilliant Violet 605™ anti-human CD25 Antibody BV605 302632 Biolegend 1:200
PE/Cyanine5 anti-human CD127 (IL-7Rα) Antibody PeCyanine 5 351324 Biolegend 1:200
PE/Fire™ 810 anti-human CD274 (B7-H1, PD-L1) Antibody PDL-1 329755 Biolegend 1:500
FITC anti-human CD34 Antibody FITC 343604 Biolegend 1:500
PE anti-human CD90 (Thy1) Antibody PE 328110 Biolegend 1:500
Pacific Blue™ anti-human/mouse CD49f Antibody Pac Blue 313620 Biolegend 1:200
Brilliant Violet 650™ anti-human CD4 Antibody BV650 317436 Biolegend 1:1000
Alexa Fluor® 647 anti-human CD8a Antibody AF647 300918 Biolegend 1:500
Brilliant Violet 421™ anti-human CD11c Antibody BV421 301628 Biolegend 1:1000
PerCP/Cyanine5.5 anti-human CD3 Antibody PerCP/Cy5.5 300328 Biolegend 1:2000
BD Horizon™ BUV661 Rat Anti-CD11b BUV661 612977 BD Biosciences 1:500
BD Horizon™ BUV563 Mouse Anti-Human CD19 BUV563 612916 BD Biosciences 1:500
BD OptiBuild™ BUV395 Mouse Anti-Human CD279 (PD-1) BUV395 745619 BD Biosciences 1:200
APC/Cyanine7 anti-human CD45RA Antibody APC-Cy7 304128 Biolegend 1:500
Spark NIR™ 685 anti-human CD62L Antibody Spark nir 685 304862 Biolegend 1:1000
APC anti-human CD38 Antibody APC 356606 Biolegend 1:2000
CD61 Armenian Hamster APC 2C9.G2 (HMβ3-1) 104316 BioLegend 1:200

Validation

Each antibody was validated for the species (mouse or human) and application (immunohistochemistry, flow cytometry) by the correspondent manufacturer. The usage was described in full detail the methods section of the manuscript.

Animals and other research organisms

Policy information about studies involving animals; ARRIVE guidelines recommended for reporting animal research, and Sex and Gender in Research

Laboratory animals

Mice were housed in individually ventilated cages with wood chip bedding plus cardboard fun tunnels and chew blocks under a 12 hour light/dark cycle at 21 ± 2°C and 55% ± 10% humidity. Standard diet was provided with ad libitum water. Mice were allowed to acclimate for at least one week in the animal facility prior to the beginning of any experiment. Adult males and females between 4-6 weeks of age were primarily used for our studies unless stated otherwise. Sample sizes were determined on the basis of a power analysis in accordance with previously published experiments. Experimenters, where necessary, were blinded to experimental groups during both scoring and quantification. The following strains were used: Rosa-Cre-ERT2 (JAX:008463), Nes-Cre- ERT2 (JAX:003771), C57BL/6 (JAX:000664), C57BL/6-Tg(Tcra2D2,Tcrb2D2)1Kuch/J (JAX:006912), B6.Cg-Tg(TcraTcrb)425Cbn/J (JAX:004194), B6.Cg-Rag2tm1.1Cgn/J (JAX:008449), B6.SJL-Ptprca Pepcb/BoyJ (JAX:002014), C57BL/6-Tg(CAG-OVAL)916Jen/J (JAX:005145), B6.Cg-Rag2tm1.1Cgn/J (JAX:008449)

Wild animals

No wild animals were used

Reporting on sex

Sex was not considered in study design, however experiments were performed on both sexes and we did not observe sex-specific effects.

Field-collected samples

This study did not involve field-collected samples

Ethics oversight

All animal work was carried out under the Animals (Scientific Procedures) Act 1986 in accordance with the UK Home office license (Project License PP9742216) and approved by the Cancer Research UK Cambridge Institute Animal Welfare and Ethical Review Board.

Note that full information on the approval of the study protocol must also be provided in the manuscript.

Clinical data

Policy information about clinical studies

All manuscripts should comply with the ICMJE guidelines for publication of clinical research and a completed CONSORT checklist must be included with all submissions.

Clinical trial registration	*Provide the trial registration number from ClinicalTrials.gov or an equivalent agency.*
Study protocol	*Note where the full trial protocol can be accessed OR if not available, explain why.*
Data collection	*Describe the settings and locales of data collection, noting the time periods of recruitment and data collection.*
Outcomes	*Describe how you pre-defined primary and secondary outcome measures and how you assessed these measures.*

Plants

Seed stocks	*Report on the source of all seed stocks or other plant material used. If applicable, state the seed stock centre and catalogue number. If plant specimens were collected from the field, describe the collection location, date and sampling procedures.*
Novel plant genotypes	*Describe the methods by which all novel plant genotypes were produced. This includes those generated by transgenic approaches, gene editing, chemical/radiation-based mutagenesis and hybridization. For transgenic lines, describe the transformation method, the number of independent lines analyzed and the generation upon which experiments were performed. For gene-edited lines, describe the editor used, the endogenous sequence targeted for editing, the targeting guide RNA sequence (if applicable) and how the editor was applied.*
Authentication	*Describe any authentication procedures for each seed stock used or novel genotype generated. Describe any experiments used to assess the effect of a mutation and, where applicable, how potential secondary effects (e.g. second site T-DNA insertions, mosiacism, off-target gene editing) were examined.*

Flow Cytometry

Plots

Confirm that:

☒ The axis labels state the marker and fluorochrome used (e.g. CD4-FITC).

☒ The axis scales are clearly visible. Include numbers along axes only for bottom left plot of group (a 'group' is an analysis of identical markers).

☒ All plots are contour plots with outliers or pseudocolor plots.

☒ A numerical value for number of cells or percentage (with statistics) is provided.

Methodology

Sample preparation

Age-matched 6-8 week-old EPZFTA-RELA and NestinCreERT2 mice were intravenously injected with CD45-PE 3 minutes prior to schedule 1. For blood, a single eye was removed using fine curved forceps, rupturing the retro-orbital sinus. Three drops of blood were collected into 1 mL of PBS with 0.025% heparin to prevent coagulation. Samples were kept on ice, for the entirety of collection. Blood was centrifuged at 400 x g for five minutes, and red blood cell (RBC) lysis was performed by resuspension in 1 mL of ACK lysis buffer(Quality Biological) for one minute, then 2 mL of ice-cold PBS was added, samples were centrifuged, and lysed red blood cells were aspirated from the leucocyte-containing pellet. The pellet was resuspended in fluorescence activated cell sorting (FACS) buffer (0.1 M, pH 7.4 PBS with 1% BSA and 1 mM EDTA) until use. Meningeal dura was carefully collected under a dissection microscope. Meninges and calvaria were then digested for 15 minutes at 37°C with constant agitation using 1 mL of pre-warmed digestion buffer (DMEM, with 2% FBS, 1 mg/mL collagenase D (Sigma Aldrich), and 0.5 mg/mL DNase I (Sigma Aldrich)), filtered through a 70 µm cell strainer, and enzymes neutralized with 1 mL of complete medium (DMEM with 10% FBS). An additional 2 mL of FACS buffer was added, samples were centrifuged at 400 x g for five minutes, and samples were resuspended in FACS buffer and kept on ice until use. For peripheral bone marrow, both tibia were flushed with 0.05% BSA PBS with 0.05% EDTA, filtered through 100 µm meshes and washed with 2% fetal bovine serum in RPMI and resuspended on 0.05% BSA PBS solution. The whole intact deep cerbical lymph nodes were mashed through a 70 µm cell strainer, using a sterile syringe plunger, and washed with 5 mL of FACS buffer. Deep cervical lymph node samples were then filtered through 100 µm meshes and washed with 2% fetal bovine serum in RPMI and resuspended on 0.05% BSA PBS solution. Brains were macrodissected based on TdTomato fluorescent signal to harvest the tumour and region-matched brain in non-tumour bearing animals. Tumour/brain samples were mechanically dissociated using sterile surgical scalpels into ~1 mm3 cubes, and dissociated using the mouse tumour dissociation kit (Miltenyi Biotec) using the gentleMACS Octo Dissociator (Miltenyi Biotec). After dissociation, the samples were then filtered through 100µm meshes and washed with 2% fetal bovine serum in RPMI, spun down 420 g for 5 minutes. Samples were resuspended in 40% percoll and centrifuged at 600 x g for 10 minutes. Supernatant was removed and washed with 2% fetal bovine serum in RPMI and resuspended on 0.05% BSA PBS solution. Samples were stained with DAPI (0.2 µg/ml). Samples were centrifuged, resuspended in FACS buffer with anti-CD16/32 (FC block; Biolegend) diluted 1:50 in FACS buffer. Cell surface stains, diluted appropriately in FACS buffer, were then added for 30 minutes on ice. For intracellular staining, suspensions were fixed and permeabilized using the Transcription Factor Fixation/Permeabilization Kit (eBioscience) per the manufacturer's instructions. Antibodies against intracellular proteins, diluted in permeabilization buffer, were added for 30 minutes on ice.

Instrument	Flow cytometry was performed using an Aurora spectral flow cytometer (Cytek Biosciences, CA, USA).
Software	Data were collected on SpectroFlo (v2.2.0.3; Cytek) and analyzed with FlowJo (v10; BD Biosciences, NJ, USA).
Cell population abundance	For each individual experiment, single-cell suspensions were incubated with viability dyes. Positive populations were gated based on negative control staining. In general, populations are given as a percentage of live, CD45+ cells.
Gating strategy	CD45− cells Zombie NIR−, CD45−<br>Myeloid cells Zombie NIR−, CD45+, CD11B+<br>Lymphoid cells Zombie NIR−, CD45+, CD11B−<br>Ly6Chi monocytes: Live/CD45+/CD11b-/Ly6G-/CD19-/TCR-b+/CD4+/GATA3+<br>Neutrophils Zombie NIR−, CD45+, CD11B+, Ly6G+<br>Monocyte-derived macrophages (MDMs) Zombie NIR−, CD45+, CD11B+, Ly6G- CD49Dhigh<br>Microglia (MG) Zombie NIR−, CD45+, CD11B+, CD49Dlow P2RY12+, CX3CR1+<br>Dendritic cells (DCs) Zombie NIR−, CD45+, CD11B+, CD49Dmed, MHC-II+, CD11C+<br>B cells Zombie NIR−, CD45+, CD11B−, CD19+, CD3−<br>– Zombie NIR−, CD45+, CD11B−, CD19−, CD3−<br>CD3+ T cells Zombie NIR−, CD45+, CD11B−, CD19−, CD3+<br>gd T cells Zombie NIR−, CD45+, CD11B−, CD19−, CD3+, gdTCR<br>NK cells Zombie NIR−, CD45+, CD11B−, CD19−, CD3−, CD161+<br>– Zombie NIR−, CD45+, CD11B−, CD19−, CD3+, CD4+, CD8−<br>Double-negative T cells (DNTs) Zombie NIR−, CD45+, CD11B−, CD19−, CD3+, CD4−, CD8−<br>CD8+ T cells Zombie NIR−, CD45+, CD11B−, CD19−, CD3+, CD4−, CD8+<br>CD4+ T cells Zombie NIR−, CD45+, CD11B−, CD19−, CD3+, CD4+, CD8−, CD25−<br>Regulatory T cells (Tregs) Zombie NIR−, CD45+, CD11B−, CD19−, CD3+, CD4+, CD8−, CD127low, CD25+ Foxp3+<br>Haematopoietic stem progenitor cells (HSPCs) Zombie NIR−, CD45+, Lineage-, Sca1+, C-Kit+<br>LS-K cells: Zombie NIR−, CD45+, Lineage-, Sca1-, C-Kit+<br>MPP2 cells: Zombie NIR−, CD45+, Lineage-, Sca1+, C-Kit+, CD150+, CD48+<br>MPP3/4 cells: Zombie NIR−, CD45+, Lineage-, Sca1+, C-Kit+, CD150-, CD48+<br>HSCs: Zombie NIR−, CD45+, Lineage-, Sca1+, C-Kit+, CD150+,CD48-, CD34+<br>GMP: Zombie NIR−, CD45+, Lineage-, Sca1-, C-Kit+, CD16/23+, CD150-<br>CFU-E: Zombie NIR−, CD45+, Lineage-, Sca1-, C-Kit+, CD16/23-, CD150-, CD105+<br>PreCFU-E: Zombie NIR−, CD45+, Lineage-, Sca1-, C-Kit+, CD16/23-, CD150+, CD105+<br>Pre-GM: Zombie NIR−, CD45+, Lineage-, Sca1-, C-Kit+, CD16/23-, CD150-, CD105-<br>PreMegEZombie NIR−, CD45+, Lineage-, Sca1-, C-Kit+, CD16/23-, CD150+ CD105-<br>Th17: Live/CD45+/CD11b-/Ly6G-/CD19-/TCR-b+/CD4+/Ror-gt+/Foxp3-<br>Treg: Live/CD45+/CD11b-/Ly6G-/CD19-/TCR-b+/CD4+/Foxp3+<br>Th1 : Live/CD45+/CD11b-/Ly6G-/CD19-/TCR-b+/CD4+/T-bet+<br>Th2: Live/CD45+/CD11b-/Ly6G-/CD19-/TCR-b+/CD4+/GATA3+

☒ Tick this box to confirm that a figure exemplifying the gating strategy is provided in the Supplementary Information.
